# Supplementary material for: miRNA Signatures in Alveolar Macrophages Related to Cigarette Smoke: Assessment and Bioinformatics Analysis
Source: Int J Mol Sci. 2025 Feb 1;26(3):1277. doi: 10.3390/ijms26031277 (PMC11818525; doi:10.3390/ijms26031277)
Supplement: Supplementary file 1 [file ijms-26-01277-s001.zip › ijms-3412562-supplementary.pdf]

miRNA Signatures in Alveolar Macrophages Related to Cigarette Smoke: Assessment and Bioinformatics Analysis

Davida Mirra [1], Renata Esposito [1], Giuseppe Spaziano [1,*], Concetta Rafaniello [2,3], Francesca Panico [4], Antonio Squillante [5], Maddalena Falciani [6], Diana Marisol Abrego-Guandique [4], Eleonora Caiazzo [1], Luca Gallelli [7], Erika Cione [8] and Bruno D'Agostino [1]

1 Department of Environmental Biological and Pharmaceutical Sciences and Technologies, University of Campania "Luigi Vanvitelli", 81100 Caserta, Italy; davida.mirra@unicampania.it (D.M.); renata.esposito@unicampania.it (R.E.); eleonora.caiazzo@studenti.unicampania.it (E.C.); bruno.dagostino@unicampania.it (B.D.)
2 Campania Regional Centre for Pharmacovigilance and Pharmacoepidemiology, 80138 Naples, Italy; concetta.rafaniello@unicampania.it
3 Section of Pharmacology "L. Donatelli", Department of Experimental Medicine, University of Campania "Luigi Vanvitelli", 80138 Naples, Italy
4 Science of Health Department, School of Medicine, University of Catanzaro, 88100 Catanzaro, Italy; francesca.panico@studenti.unicz.it (F.P.); dianamarisol.abregoguandique@unicz.it (D.M.A.-G.)
5 Department of Medicine, University of Salerno, 84100 Salerno, Italy; a.squillante92@gmail.com
6 Pulmonary and Critical Care Medicine, Ospedale Scarlato, 84018 Scafati, Italy; m.falciani@aslsalerno.it
7 Clinical Pharmacology and Pharmacovigilance Unit, Department of Health Sciences, Mater Domini Hospital, University of "Magna Graecia", 88100 Catanzaro, Italy; gallelli@unicz.it
8 Department of Pharmacy, Health and Nutritional Sciences, University of Calabria, 87036 Rende, Italy; erika.cione@unical.it
* Correspondence: giuseppe.spaziano@unicampania.it; Tel.: +39-(0)-823-274-623

1. Materials and Methods

1.1. Study Population and Bronchoalveolar lavage

This study belongs to a cross-sectional nonpharmacological clinical study recorded at clinicaltrials.gov (NCT04654104) and all procedures and protocols described were approved by the local Ethics Committee "Calabria Centro". The criteria of the Institutional Review Board/Human Subjects Research Committee, the Declaration of Helsinki, and the Guidelines for Good Clinical Practice were followed and, all patients or legal guardians signed an informed consent form prior of the beginning of the study. We enrolled 43 individuals who were equally distributed in terms of age (≥18 years) and sex, at the "Mater Domini" Hospital in Catanzaro, Italy. All participants underwent spirometry in compliance with international guidelines as well as bronchoscopy and BAL for suspected pulmonary neoplasia [1]. Samples that were not employed for histopathological purposes or in our previous research were used in the current study [2]. Based on the clinical data and the pathological diagnosis obtained after bronchoscopy, we divided the enrolled subjects into:1) healthy never-smoker control ("HNS"; n = 9); 2) healthy ever-smokers' control ("HS"; n = 11); 3) smokers with Global Initiative for Obstructive Lung Diseases (GOLD) stage 1–4 ("COPD," n =11); 4) non-small cell lung cancer ("NSCLC"; n=12). The main clinical and pathological characteristics of the cohorts are reported in our previous [2]. In summary, those who had lung infections, extrapulmonary tumors, airflow obstruction other than COPD, autoimmune disorders, or who did not sign the informed consent form were excluded. All enrolled subjects were smokers except for HNS group; specifically,

HS were 8 current and 3 former smokers, COPD were 11 current smokers and NSCLC were equally distributed between current and ex-smokers. Within each group, the subjects were comparable in terms of age, sex, and lung cancer histology. Indeed, only those with NSCLC were enrolled among the subject's presenting cancer. The most frequent comorbidities were hypertension ($p < 0.05$), and the most used drugs were bronchodilators ($p < 0.0001$). After obtaining informed consent, the subjects underwent standard flexible bronchoscopy for clinical indications [1]. Premedication and local anesthesia were followed by BAL with 200 ml of sterile isotonic saline solution (37 °C) in the right middle lobe. Specifically, BAL was obtained by instilling 50 ml up to four times, as previously reported [2]. The samples were filtered through sterile gauze and centrifuged at 400 g for 10 min at 4 C° to pellet cellular material. The cells were washed, resuspended in buffer phosphate saline (PBS), and counted in a Bürker chamber. The cell yield was determined as the total cells /total volume obtained for each saline installation. Then, cell viability was determined by Trypan blue exclusion assay, and differential cell count was performed with QUICK-DIFF staining; at least 100 cells were counted.

1.2. Preparation of CS extract and cell culture procedures

CS extract was prepared as previously described bubbling ten Red Marlboro cigarettes (Phillip Morris; Cracow, Poland) without filter through 250 ml of serum-free RPMI with a customized vacuum pump apparatus. The obtained suspension was adjusted to pH 7.4 and filtered through a 0.20 μm pore filter to remove bacteria and large particles. Macrophages from acute monocytic leukemia (THP-1) were used as a pilot model to establish the exact dose (2%, 5%, or 10%) of CS that was able to induce metabolic impairment at 24h using the Thiazolyl Blue Tetrazolium Bromide solution (MTT) assay. THP-1 cells (ATCCR TIB-202TM), purchased from the American Type Culture Collection (Manassas, Virginia, USA), were maintained at 2×10^5 cells/ml in RPMI-1640 medium containing 10% FBS and 2 mM L-glutamine, 200 U/ml penicillin, and 200 mg/ml streptomycin. To obtain a macrophages-like phenotype, THP-1 cells were treated with 100 ng/ml phorbol 12-myristate 13-acetate (PMA, Sigma-Aldrich) for two days. The cells were then incubated with fresh medium for one day to allow cell recovery and exposed to 2%, 5%, or 10% CS medium for 24h. Following 24 h MTT was added and incubated for 4 h to perform the proliferation assay. MTT is a colorimetric method that allows to assess the mitochondrial reductive function as an indicator of growth inhibition. After 4 h, DMSO was added to measure the absorbance at 570 nm using a microplate reader. In another set of experiments, the cells were seeded at a density of 5×10^4 per well in 24-well plates on 18 mm coverslips, PMA-differentiated and treated with CS extract for 24h, as previously described. Then, the cells were fixed and stained with QUICK-DIFF solution to highlight cell morphology.

BAL cell pellets were suspended in RPMI-1640 medium supplemented with 10% FBS, 2 mM L-glutamine, 200 U/ml penicillin, and 200 mg/ml streptomycin. The cell suspension was added at 0.5×10^6 cells/mL to a 75-tissue culture flask and maintained at 37 °C in a 5% CO2 humidified milieu for 2 h to allow AMs adherence. Lymphocytes, red blood cells and other non-adherent cells were removed by washing several times with PBS. AMs purity, as determined by morphology, was greater than 95%. To confirm the results obtained in TH-P1 cells, we performed an additional MTT assay in AMs obtained from three healthy never smokers doners. The AMs were then exposed to 10% CS for 24 h, based on previous treatment result.

1.3. Biochemistry Assays and Real Time PCR (RT-PCR)

The extraction of miRNAs in AMs obtained from BAL was carried out through the miRNeasy mini kit and RNA was eluted at a volume of 15 μL, as previously described [2]. RNA degradation was assessed using a qubit RNA Integrity and Quality (IQ) assay (catalog number Q33222) with a Qubit 4 fluorometer (serial number 2322618032114). The expression levels of has-miR-34a-5p, 17-5p, 16-5p, 106a-5p 223-5p and 20a-5p were determined using TaqMan™ Advanced miRNA

Assay RT-PCR, following Thermo Fisher Scientific procedures (Waltham, MA, USA), with U6 snRNA as the housekeeping miRNA as previously described [2]. Nine biological replicates for the HNS group, eleven for HS, eleven for COPD, and twelve for NSCLC were analyzed, and all samples were run in triplicate; after the achievement of the RT-PCR, the cycle threshold (Ct) of the reactions was determined. Data from all RT–PCR experiments and miRNA expression was analyzed applying the comparative and normalizing to the endogenous miRNA control $2^{\wedge(-DDCt)}$ method, where DCt $= Ct_{miRNA} - Ct_{housekeeping\ miRNA}$, whereas the relative differences in expression was determined with DDCt = $DCt_{HS/COPD/NSCLC\ (with\ or\ without\ CS)} - DCt_{HNS}$.

1.4. Bioinformatic analysis

mRNA targets of has-miR-34a-5p, 17-5p, 16-5p, 106a-5p 223-5p and 20a-5p linked to inflammation or AMs properties were analyzed by DIANA Tools and miRpath v3. Then, for the CS focused sub-analysis, multimiR package that integrates miRecords, miRTarBase and Tarbase [3] was utilized to identify the validated target genes associated with differentially expressed miRNAs using genes common in at least two databases for subsequent analysis [4]. Clusterprofiler R packages were used to perform KEGG pathways enrichment analyses with a specific focus on exploring the relationship between genes targeted by differential miRNAs identified through RT-PCR analysis and CS-related. In addition, the Gene Ontology (GO) enrichment analysis parameters for Biological Process (BP), Cellular Component (CC), and Molecular Function (MF) were obtained, and three in-one bar plots created by the Enrichplot. Finally, the STRING website was utilized to identify the protein-protein interaction (PPI) and to generate Reactome Pathways Enrichment [5]. Subsequently, the interaction networks at the gene level were built by the Cytoascape v. 3.9.1 software. Finally, the clustering procedure was also performed using the Molecular Complex Detection (MCODE) algorithm version 2.0.2 [5]. The most relevant nodes were selected based on biological relevance, metrics such as betweenness centrality, closeness centrality, and topological coefficient. Consideration was given to interactions with a medium confidence score > 0.4.

1.5. Statistical Analysis

Unless specified, all data are expressed as mean ± standard deviation (SD). The ordinary one-way ANOVA test followed by Dunnet Multiple Comparison Test with a single pooled variance was used for MTT assay. The ordinary one-way ANOVA test followed by Tukey Multiple comparison test with a single pooled variance was used to assess the differences in miRNAs expression between AMs exposed or not to CS in each group. Nominal (sex, age, comorbidity, or treatment) and categorical variables were considered and the correlation between clinical data was calculated using one-way ANOVA followed by Tukey Multiple Comparison Test. GraphPad software (version 9.1.0) was used for statistical analyses (GraphPad Software, San Diego, CA, USA). Differences were considered statistically significant at $p < 0.05$.

2. Results

2.1. In silico identification of target mRNAs

The relationship between miRNAs and lung response to CS was assessed by in silico analysis of sequence similarity between miRNAs and different mRNAs. A total of 12,186 experimentally validated target genes were compared in miRPath. We analyzed all selected miRNA targets, focusing on those that are common to several miRNA and implicated in the cellular pathways regulating inflammation. The results showed that miRNA may be involved in regulation of 740 genes involved in inflammation pathways, such as BCL2, MTOR, MCL1, TGFBR, SMAD or VEGF, among others. However, other target genes linked to apoptosis or cytokines production were found. Given that several authors have experimentally confirmed all genes, miRNA might modulate these targets in a coordinated or individual manner, affecting several hallmarks of lung response to CS.

microRNAs are related to inflammation pathways

Sixty-two pathways were significantly enriched ($p < 0.05$). Figure 4 shows the identified 20 KEGG-enriched pathways associated with validated targets of the comprehensive miRNAs related to inflammation pathways, such as MAPK signaling pathways, focal adhesion, cell cycle, endocytosis, TNF signaling pathways, TGF-beta and VEGF signaling pathways, among others.

Protein-protein interaction (PPI)

To better understand the interactions among experimental validated targets of the miRNAs regulated by CS, PPI networks were constructed. It was created one PPI network with 740 nodes and 22814 edges was obtained, and the average number of neigh-bours was 61.6. The Molecular Complex Detection (MCODE) generated sub-network significant. The first network was obtained with 89 nodes and 3126 edges, and the average number of neighbours was 70.2. The nodes include TGFB1, PTEN, SMAD4, MCL1, FOXO1, NFKB1, IL6, mTOR and SIRT1 (Figure 5A). The second network with score 15.78 was obtained, including 57 nodes such as VEGFC, ILA, TGFBR2 and SMAD7 (Figure 5B) and the third module with score 11.60 and 92 nodes and 528 edges, including IL6R, VEGFB and VEGFD (Figure 5C).

2.2. CS focused sub-analysis

We performed further bioinformatic analysis focused on has-miR-106a-5p, 17-5p, 20a-5p, and 223-5p 34a-5p, which exhibit CS-dependent modulation. KEGG analysis showed that cellular senescence exhibited the highest enrichment score (−log 10 (p value) ≈10) (Figure 7). Similarly, the p53 signalling pathway, a key regulator of the cell cycle and apoptosis, showed a high enrichment score and significance (−log 10 (p-value) ≈8) (Figure 7). Figure S1 shows Gene ontology (GO) analysis of the target genes. Among the biological processes dataset, protein localization to the nucleus and miRNA transcription had high enrichment scores (−log 10 (p value) ≈10); focal adhesion and protein kinase complex were the most statistically significant among cellular components (−log 10 (p value) ≈7), whereas ubiquitin protein ligase binding and SMAD binding (−log 10 (p value) ≈10 and −log 10 (p value) ≈10, respectively) were the most enriched among molecular functions. Figure S2 shows the PPI network with 380 nodes and 3960 edges, highlighting the hierarchical nature of protein interactions, with a few highly connected hubs (purple nodes) surrounded by less-connected proteins (yellow nodes). MCODE allowed us to obtain a subnetwork with 35 nodes and 390 edges (A) and one with 25 nodes and 134 edges (B) (Figure 8). TP53, ATM, and MYC from the first subnetwork and ESR1, BRCA1, and SMAD4 from the second appear to be central, suggesting that they may play key roles in the network. Finally, Reactome Pathway enrichment identifies the signalling pathways and biological processes in which the target proteins are involved. The most

represented pathways, indicated by the largest bubble, were the signal transduction pathways, with the highest gene count in the dataset, while the most enriched one was the TGFB family members signalling pathway, suggesting a central role of miRNAs in key biological processes (Figure 9).

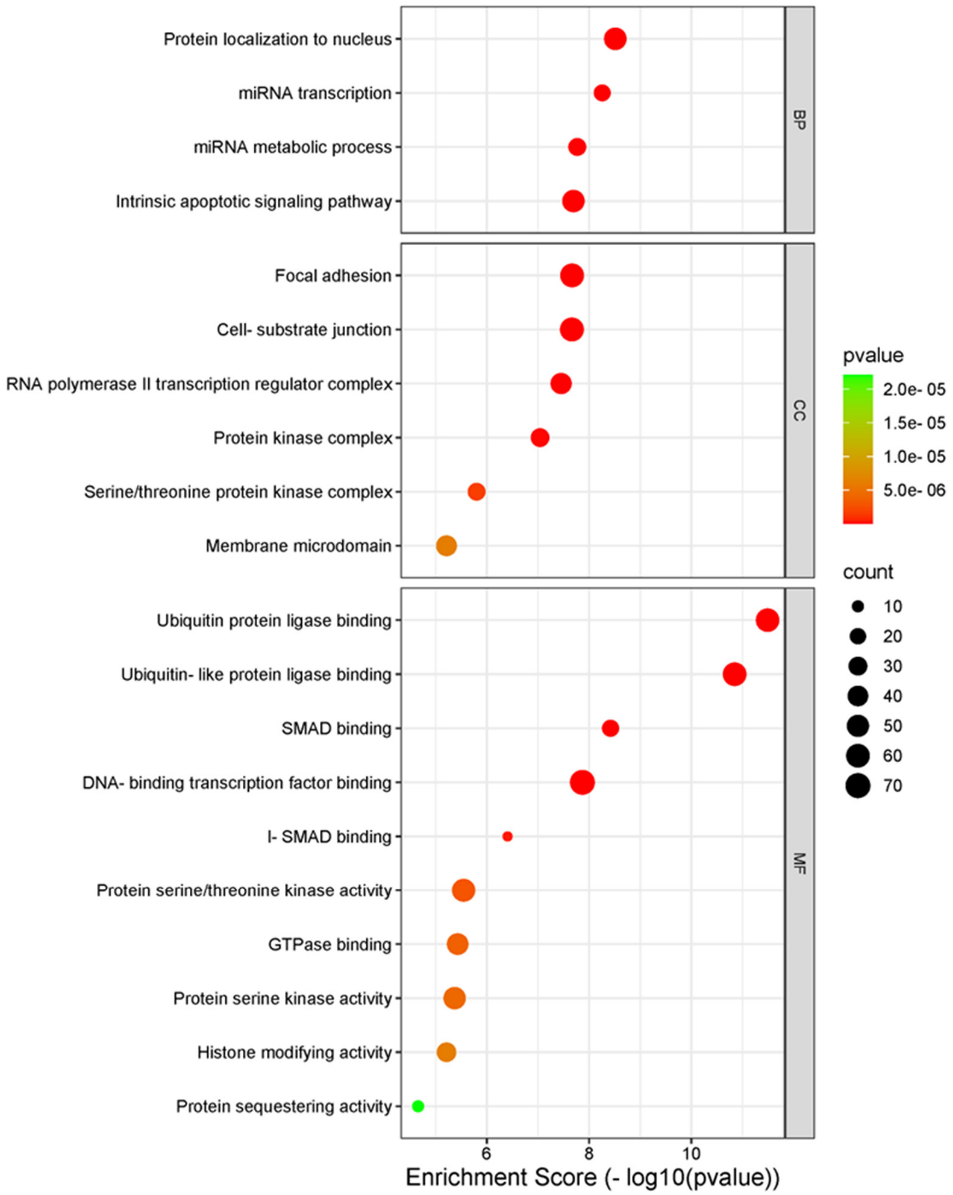

Figure S1. Gene ontology (GO) analysis of target genes, the colour of each dot represents the p-value of each term involved in the analysis. The size of each dot represents the counts of overlapped genes between the input genes and the total gene list on GO. BP, biological processes; CC, cellular component; MF, molecular function.

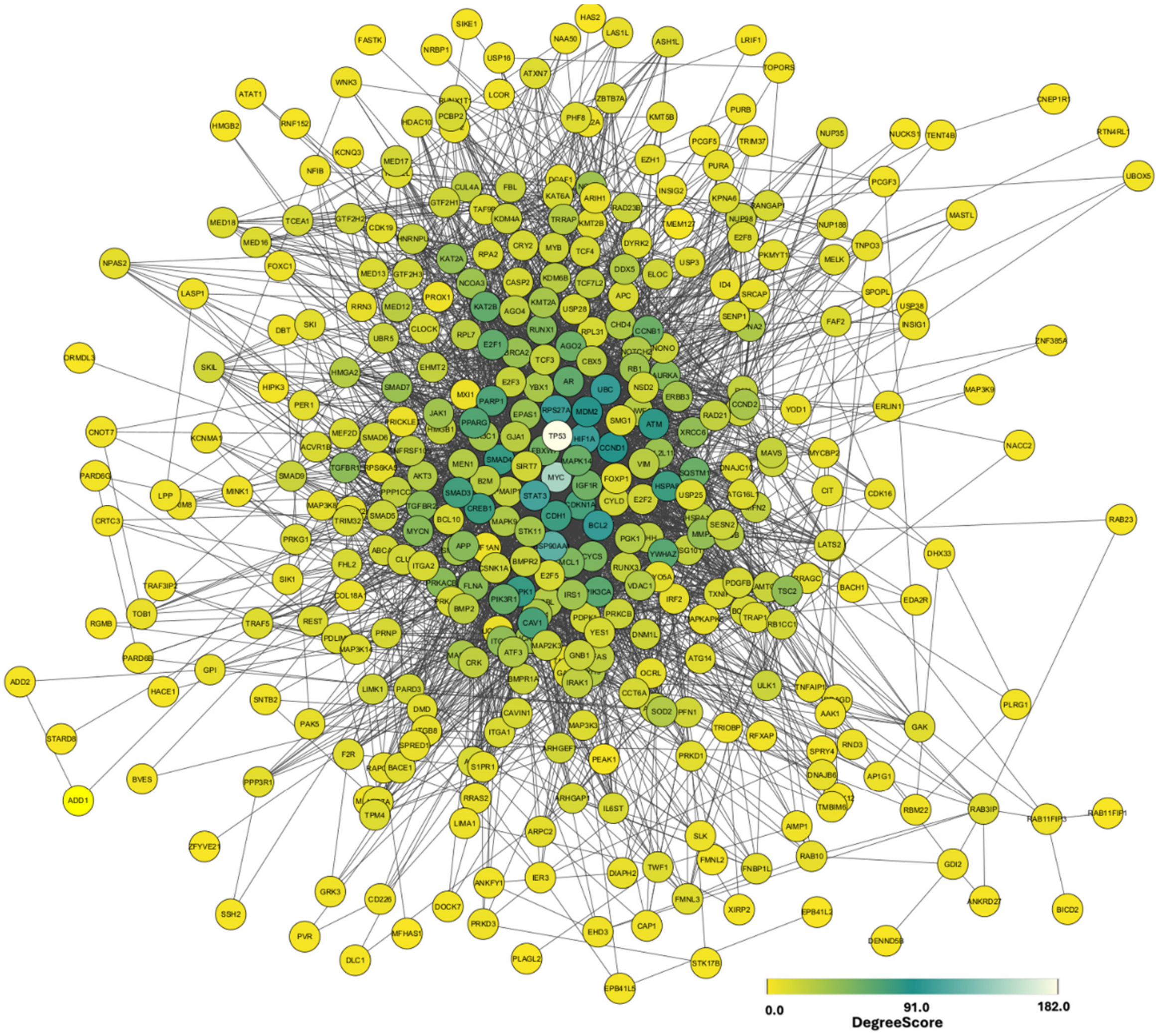

Figure S2. Protein-protein interaction (PPI) network with 380 nodes and 3960 edges and principal centralities identified in our study (based on targets present in gene ontology analysis). The size of nodes clarifies the Degree, and node color clarifies Betweenness.

References

1. Chen, H.; Luo, Y.; Lin, M.; Peng, X.; Liu, M.; Wang, Y.; Li, S.; Yang, D.; Yang, Z. Serum Exosomal MIR-16-5p Functions as a Tumor Inhibitor and a New Biomarker for PD-L1 Inhibitor-dependent Immunotherapy in Lung Adenocarcinoma by Regulating PD-L1 Expression. Cancer Med. **2022**, 11, 2627–2643.

2. Mirra, D.; Esposito, R.; Spaziano, G.; Sportiello, L.; Panico, F.; Squillante, A.; Falciani, M.; Cerqua, I.; Gallelli, L.; Cione, E.; et al. MicroRNA Monitoring in Human Alveolar Macrophages from Patients with Smoking-Related Lung Diseases: A Preliminary Study. Biomedicines 2024, 12, 1050.

3. Ru, Y.; Kechris, K. J.; Tabakoff, B.; Hoffman, P.; Radcliffe, R. A.; Bowler, R.; Mahaffey, S.; Rossi, S.; Calin, G. A.; Bemis, L.; Theodorescu, D. The multiMiR R package and database: integration of microRNA-target interactions along with their disease and drug associations. Nucleic acids research 2014, 42, e133.

4. Szklarczyk, D.; Gable, A. L.; Nastou, K. C.; Lyon, D.; Kirsch, R.; Pyysalo, S.; Doncheva, N. T.; Legeay, M.; Fang, T.; Bork, P.; Jensen, L. J.; von Mering, C. The STRING database in 2021: customizable protein-protein networks, and functional characterization of user-uploaded gene/measurement sets. Nucleic acids research 2021, 49(D1), D605–D612.

5. Bader, G.D., Hogue, C.W. An automated method for finding molecular complexes in large protein interaction networks. BMC Bioinformatics 2003, 4, 2.
